# Food Insecurity, Nutritional Status, and Factors Associated with Malnutrition among People Living with HIV/AIDS Attending Antiretroviral Therapy at Public Health Facilities in West Shewa Zone, Central Ethiopia

**DOI:** 10.1155/2018/1913534

**Published:** 2018-05-06

**Authors:** Delelegn Yilma Gebremichael, Kokeb Tesfamariam Hadush, Ermiyas Mulu Kebede, Robel Tezera Zegeye

**Affiliations:** ^1^College of Medicine and Health Sciences, Department of Public Health, Ambo University, P.O. Box 19, Ambo, Ethiopia; ^2^College of Medicine and Health Sciences, School of Medicine, Addis Ababa University, Addis Ababa, Ethiopia

## Abstract

**Background:**

In resource limited settings, HIV/AIDS patients lack access to sufficient nutritious foods, which poses challenges to the success of antiretroviral therapy. HIV/AIDS and malnutrition are still major public health problems in Ethiopia. Though measuring nutritional status is an essential part of ART program, little evidence exists on food insecurity and nutritional status of HIV/AIDS patients in Ethiopia. Hence, the study aimed to determine food insecurity and nutritional status and contextual determinants of malnutrition among HIV/AIDS patients in West Shewa Zone, Ethiopia.

**Methods:**

Institution-based cross-sectional study was conducted among HIV/ADIS patients who have been attending antiretroviral therapy at public health facilities in West Shewa Zone from April to May 2016, Ethiopia. The sample size was 512 and study participants were selected from each facilities using systematic random sampling method. Data were collected using pretested questionnaire by trained data collectors. Data were entered to Epi-Info 3.5.1 for Windows and analyzed using SPSS version 22. Logistic regression analyses were conducted to determine independent factors associated with malnutrition.

**Results:**

Prevalence of malnutrition was 23.6% (95% CI: 19.7%–27.4%) and prevalence of household food insecurity was 35.2% (95% CI: 31.1%–39.0%). Factors significantly associated with malnutrition among HIV/AIDS patients were unemployment (AOR = 3.4; 95% CI: 1.8–5.3), WHO clinical stages III/IV (AOR = 3.3; 95% CI: 1.8–6.5), CD4 count less than 350 cells/*μ*l (AOR = 2.0; 95% CI: 1.8–4.2), tuberculosis (AOR = 2.3; 95% CI: 1.3–4.9), duration on antiretroviral therapy (AOR = 1.8; 95% CI: 1.2–2.9), and household food insecurity (AOR = 5.3; 95% CI: 2.5–8.3).

**Conclusions:**

The findings revealed high prevalence of malnutrition and household food insecurity among HIV/AIDS patients attended ART. The negative interactive effects of undernutrition, inadequate food consumption, and HIV infection demand effective cross-sectorial integrated programs and effective management of opportunistic infections like tuberculosis.

## 1. Background

The burden of HIV/AIDS remains unacceptably high in low and middle income, particularly in Africa [1]. In resource limited settings, many PLWHA lack access to sufficient quantities of nutritious foods, which poses additional challenges to the success of antiretroviral therapy (ART) [2, 3]. The combined impacts of food insecurity and HIV/AIDS place further strain on already limited household resources as affected family members struggle to meet household food needs [4]. Malnutrition and food insecurity are associated with increased mortality and poor clinical outcomes among people living with HIV/AIDS [5]. The nutritional problems have been shown to be significant and contribute to morbidity and mortality in HIV+/AIDS patients [6]. Evidence indicates that even relatively small losses in weight (5%) are associated with decreased survival rate [7]. There is an urgent need for renewed focus on and use of resources for nutrition as a fundamental part of the comprehensive package of care at country level [8, 9].

People living with HIV (PLWHA) are more likely to become malnourished due to reduced food intake, poor absorption of nutrients, and changes in the way the body uses nutrients it receives or has stored [10]. In Ethiopia, undernutrition prevalence was reported from 12.3% [11] to 46.8% [12] among PLWHA and high prevalence of food insecurity among PLWHA was reported from 40.4% [13] to 87.4% [14]. Food insecurity and undernutrition are still significant problems among PLWHA on ART in Ethiopia despite advances in their treatment and survival [12–17]. However, little is known about contextual factors associated with the complex interactions of food insecurity and malnutrition among PLWHA in Ethiopia. Hence, this study intended to assess contextual factors of undernutrition among PLWHA because understanding the relationships between HIV, undernutrition, and food insecurity has important implications for the effective integration of nutritional interventions into HIV programs.

## 2. Materials and Methods

The study was conducted in West Shewa Zone which is one of the Zones in Oromia region located 114 kilometers west of capital city of Ethiopia. According to 2015 West Shewa Zone report, 18,642 patients were ever enrolled, 10,988 were ever started ART, and 8006 were on ART in West Shewa Zone. There are 3 hospitals and 83 health centers that currently provide protective, promotive, preventive, curative, and rehabilitative services. Among 83 facilities, 24 of them provide antiretroviral treatment (ART) services in West Shewa Zone. The study used institution-based cross-sectional study design. The source population was people living with HIV attending antiretroviral therapy at public health facilities in West Shewa Zone and the study population was HIV/ADIS patients aged 18 years or older who have been attending ART at public health facilities in West Shewa Zone from April to May 2016, Ethiopia.

Sample size for this study was determined using the formula for the estimation of single proportion. *p* = proportion of HIV positive individuals with malnutrition taken as 28% [15], *d* = margin of error = 0.05, and *Z* = value of standard normal distribution (*Z* statistic) at 95% confidence level (*z* = 1.96). After being multiplied by 1.5 design effect and adding 10% nonresponse rate, the final sample was 512. Among 24 health facilities which provide ART services in West Shewa Zone, six health facilities were randomly selected using lottery method and the samples were proportionally allocated to each of the selected health facilities. Then the study participants were selected from each of the health facilities using systematic random sampling method.

Clinical, laboratory, and ART data were collected by trained health professionals through reviewing records from ART entry registration book and individual follow-up form using prepared pretested data collection form. Household food insecurity was assessed using Household Food Insecurity Access Scale (HFIAS) [18]. It consists of nine questions on experiences of food insecurity, with possible responses of never, rarely, sometimes, or often. The highest score for HFIAS is 27; the higher the score, the greater the food insecurity. Food insecurity is considered when the individual (PLHIV) answer >2 to affirmative questions of food insecurity scale resulting from financial resource constraint. The height and weight of the patients were measured in light clothing and bare foots calibrated to 0.5 cm and 0.5 kg, respectively. Height was measured while the patients were standing erect in a Frankfurt position and the weight was measured on a standing scale. BMI (Weight (kg)/Height2 (m)) was calculated to assess nutritional status. The standard cutoffs were used to define nutritional status. If individual's BMI was less than 18.5 kg/m2, the individual was considered undernourished/malnourished; when it was greater than or equal to 25.0 kg/m2, the individual was considered overweight; and if it was 18.5–24.9 kg/m2, the individual was considered normal.

Data were checked for completeness and consistency, coded, and entered to Epi-Info 3.5.1 for Windows and analyzed using SPSS version 22 for Windows. Study participants' characteristics were described in terms of mean (standard deviation) and median (interquartile range) for continuous data and frequency distribution for categorical data. Chi square (*χ*^2^) test was used for categorical variables. Multicollinearity between explanatory variables was checked using variance inflation factors (VIF). Logistic regression analysis was carried out to identify the correlates of nutritional status. All explanatory variables that were significantly associated with the outcome variable in the bivariate analyses (*P* < 0.05) were entered in to multivariate logistic regression model. Crude and adjusted odds ratios with their 95% confidence interval (CI) were determined and statistical significant association was asserted based on *P* value less than 0.05.

Ethical clearance was obtained from the Institutional Review Board (IRB) of Ambo University. Following the approval by IRB, official letter of cooperation was written to the concerned bodies from the College of Medicine and Health Sciences research office, about the purpose of the study to facilitate the support and commitment of responsible bodies. Written consent from each of the study subjects was taken. Study participants' confidentiality was maintained. No personal identifiers were used on data collection forms and the recorded data were not accessed by a third person, except the Principal Investigators.

## 3. Results

### 3.1. Sociodemographic Characteristics of People Living with HIV/AIDS

A total of 505 study participants responded to the study with response rate of 98.6%. The mean age (SD) was 34.41 (±7.47) years and 368 (72.9%) of the study participants were in 25 to 44 years' age group. More than half of the study participants, 264 (52.3%) were females. Majority of the study participants, 350 (69.3%) were rural resident and 337 (66.7%) were married. Out of the total respondents, 417 (82.6%) were Oromo in ethnicity and 299(59.2%) were Orthodox Christians. Regarding education, 324 (64.2%) respondents were literate and 423 (83.8%) of participants were employed (Table 1).

### 3.2. Clinical Characteristics and Household Food Security Status of People Living with HIV/AIDS

The patients median (IQR) duration of ART treatment was 44 (22–68) months. Nearly half (47.7%) of the study participants were at clinical WHO stage III and a quarter (25.4%) were at clinical WHO stage IV. Baseline median CD4 count (IQR) was 135 (68–211) cells/*μ*l. The most recent median CD4 count (IQR) was 232 (134–328) cells/*μ*l. More than three-quarters (76.8%) of the study participants had CD4 count less than 350 cells/*μ*l. The history of TB/HIV coinfection and anemia was 12.5% and 20.6%, respectively. The prevalence of household food insecurity was 35.2% (95% CI: 31.1%–39.0%) (Table 2).

### 3.3. Nutritional Status of People Living with HIV/AIDS

Concerning nutritional status, 23.6% (95% CI: 19.7%–27.4%) of respondents were undernourished. The result showed that undernutrition was significantly different for men (18.7%) compared to women (28%) (*P* value = 0.009). Malnutrition was higher among widowed (33.3% respondents compared with married participants (21.9%), *P* value = 0.032). There was also significant difference in malnutrition status between literate (19.8%) and illiterate respondents (30.4%) (*P* value = 0.004). Similarly, unemployed respondents had significantly higher undernutrition (40.2%) compared with employed counterparts (20.8%) (*P* value = 0.0001).

Malnutrition was also significantly different with WHO clinical stages (37.5%, 24.1%, 10.3%, and 8.2% of stage IV, stage III, stage II, and stage I patients, resp., were undernourished, *P* value = 0.0001). PLWHA who had CD4 count less than 350 cells/*μ*l had significantly higher malnutrition (27.6%) compared with PLWHA who had CD4 count greater than 350 cells/*μ*l (10.3%) (*P* value = 0.003). TB/HIV coinfected patients had significantly higher undernutrition (39.7%) compared to their counterparts (21.3%), *P* value = 0.002. The prevalence of undernourishment was higher in PLWHA who were on ART for less than 44 months (29.9%) than those who were on ART for more than 44 months (16.8%) (*P* value = 0.004). Likewise, household food unsecured PLWHA (48.9%) were significantly undernourished higher than household food secured counterparts (9.8%) (*P* = 0.0001) (Table 3).

### 3.4. Factors Associated with Nutritional Status among HIV/AIDS Patients

The independent factors significantly associated with nutritional status among people living with HIV/AIDS were occupation, WHO clinical stage, CD4 count, tuberculosis, duration on ART, and household food security status. Unemployed PLWHA were 3.4 more likely to be undernourished compared with employed counterparts, AOR = 3.4 (95% CI: 1.8–5.3). WHO clinical stages III and IV were found to be significant factors associated with under nutrition; PLHIVA in stage III and IV were 3.3 times more likely to be undernourished than WHO stages I and II, AOR = 3.3 (95% CI: 1.8–6.5). Similarly, CD4 count was a significant factor associated with undernutrition. PLHIVs with CD4 counts of less than 350 cells/*μ*l were 2 times more likely to be undernourished than their counterparts, AOR = 2.0 (95% CI: 1.8–4.2). Previous OIs with tuberculosis were also found to be the prominent risk factor for undernutrition; TB/HIV coinfected patients had 2.3 significantly higher undernutrition compared to their counterparts, AOR = 2.3 (95% CI: 1.3–4.9). Duration on ART was also significantly associated with nutritional status of HIV/AIDS patients (AOR = 1.8; 95% CI: 1.2–2.9). Likewise, household access to food was the strongest factor associated with undernutrition. Household food insecure PLWHA were 5.3 times more likely to be undernourished than household food secured counterparts (AOR = 5.3; 95% CI: 2.5–8.3) (Table 4).

## 4. Discussion

This study revealed important information about nutritional status and contextual associated factors among PLWHA who have been on ART in West Shewa Zone, Ethiopia. PLWHA are more vulnerable to malnutrition than the general population and nutritional status is a good predictor of their mortality risk [5, 6]. The prevalence of undernutrition in this study (23.6%) was comparable with the findings of other similar studies [7, 15, 16, 19, 20]. Studies conducted in Ethiopia and other developing countries reported high prevalence of undernutrition among PLWHA compared to our finding [12, 13, 21, 22]. Contrarily, other studies carried out in different study settings revealed lower figures of undernutrition than the result of this study [11, 23–25]. Variation in sociodemographic characteristics of the study participants, in year when studies were conducted, in level of awareness creation made regarding HIV and nutrition, in household food security status, in nutritional support, in opportunistic infections, in severity of the disease, and in adherence to ART treatment might have attributed to divergence in the figures of undernutrition.

This study revealed that unemployed PLWHA were more likely to be undernourished compared with employed counterparts. The higher risk of developing malnutrition in unemployed subjects found in this study is supported by findings of other studies where unemployment promotes poverty, which in turn limits the ability of individual to expend money for food consumption due to low income [11, 15, 23, 26]. The implication is improving household income and creating employment opportunities for PLWHAs might be essential component in comprehensive cares for HIV/AIDS patients.

The study showed that WHO clinical stages III and IV have significant effects on the likelihood of malnutrition development among HIV/AIDS patients. Malnutrition is usually encountered at the advanced phase of the HIV infection [27] and anthropometric measurements are lower in symptomatic HIV/AIDS patients classified by WHO stages [28]. This finding was supported by a study conducted in Nepal that reported that WHO clinical stages III and IV were found to be significant risk factors of undernutrition [29]. Similarly, studies done in Ethiopia revealed WHO clinical stage four was significantly associated with malnutrition [11, 12], and study conducted in Uganda revealed that HIV/AIDS patients in WHO stage four usually characterized by severe wasting and food aid to PLWHA delayed HIV disease progress [30]. This finding also supported by a study done in Zimbabwe revealed that patients in advanced stage were more likely to be malnourished [31].

This study also indicated CD4 count was a significant factor associated with undernutrition. PLHIVA with CD4 counts of less than 350 cells/*μ*l were two times more likely to be undernourished than their counterparts. The finding was consistent with study done in Nepal [29]. It also supported by other findings in which patients with CD4 counts less than 200 cells/*μ*l were more likely to be undernourished [19, 20, 32, 33]. Evidence indicated that the HIV-induced immune impairment and increased subsequent risk of opportunistic infections can worsen nutritional status [34]. Studies also found that malnutrition was associated with lower CD4 count [5], and it tends to decrease CD4 counts recovery and predisposes patients to early death [35]. It was supported by the finding that micronutrient supplements significantly increased CD4 count among PLWHA [36].

Opportunistic infections place PLWHA at a high risk of developing malnutrition [37]. The study showed that tuberculosis (TB) among HIV/AIDS patients was an independent risk factor for undernutrition. This finding was consistent with other similar studies [16, 29, 38–41]. A study done in southern Ethiopia also found number of previous opportunistic infections (OIs) significantly associated with malnutrition [11]. Other studies conducted in Ethiopia also revealed association between malnutrition and tuberculosis [42, 43]. Evidences also stated that the HIV-induced immune impairment and opportunistic infections can worsen nutritional status [34], and tuberculosis could result in nutritional consequences that commonly precipitate appetite loss, weight loss, and wasting and significantly determine incidence and severity of nutritional status [8].

The study showed that duration of ART treatment was significantly associated with nutritional status of HIV/AIDS patients where significantly lower prevalence of undernutrition was found among patients who were on ART for longer duration. A study conducted in Tanzania also reported that the risk of wasting in HIV patients reduced as ART treatment duration increased [44]. This might be because ART treatment for long period of time improves immunity and reduces risk of opportunistic infections, diarrhea, and vomiting as a result patient could have better appetite, increased dietary intake, and improved nutritional status [44, 45].

Household access to food is a key indicator for predicting undernutrition. Prevalence of household food insecurity was 35.2%. This finding was the lowest compared to other studies in Ethiopia reported from 40.4% to 87.4% [13, 14, 17, 20, 46–48]. This variation might be as a result of difference in socioeconomical characteristics of the study populations or variation in time when the studies were conducted. The result showed that households with food insecurity were more likely to be undernourished than those PLWHA with adequate access to food. This finding was is in line with other studies [19, 31, 49]. Evidences showed that HIV/AIDS deepens food insecurity and affects the nutritional status of PLWHA leading to weight loss and wasting since PLWHA may no longer hold jobs, manufacture goods, and provide services [8, 50] or because of the decrease in productivity [5, 49]. These findings indicated that the negative interactive effects of undernutrition, inadequate food consumption, and HIV infection demand multisector interventions.

The study had limitations that should be acknowledged. This study was subject to selection bias since the respondents were ones who were actively seeking routine medical care selected from health facilities. They may not represent patients who do not come to the institution from the communities; therefore, generalizability is only for those who are on regular ART follow-up. The cross-sectional nature of the study itself limits conclusions about cause-effect temporal relationships between explanatory variables and undernutrition.

## 5. Conclusions

The findings revealed high prevalence of malnutrition and household food insecurity among people living with HIV/AIDS on antiretroviral therapy in Central Ethiopia. Factors significantly associated with undernutrition among people living with HIV/AIDS were unemployment, WHO clinical stages III/IV, CD4 count less than 350 cells/*μ*l, tuberculosis, duration on ART, and household food insecurity. The negative interactive effects of undernutrition, inadequate food consumption, and HIV infection demand effective cross-sectorial integrated programs and effective management of opportunistic infections like tuberculosis.

## Figures and Tables

**Table 1:** Sociodemographic characteristics of HIV/AIDS patients attending antiretroviral therapy in public health facilities, West Shewa Zone, Ethiopia, 2017.

Sociodemographic characteristics	Frequency	Percent (%)
Age		
18–24	38	7.5
25–34	175	34.7
35–44	193	38.2
45–54	82	16.2
>54	17	3.4
Sex		
Male	241	47.7
Female	264	52.3
Residence		
Rural	350	69.3
Urban	155	30.7
Marital status		
Single	92	18.2
Married	337	66.7
Widowed	51	10.1
Divorced/separated	25	5.0
Ethnicity		
Oromo	417	82.6
Amhara	71	14.0
Others	17	3.4
Religion		
Protestant	177	35.1
Orthodox	299	59.2
Muslim	24	4.7
Others	5	1.0
Education		
Illiterate	181	35.8
Literate	324	64.2
Occupation		
Employed	423	83.8
Unemployed	82	16.2

**Table 2:** Clinical characteristics of HIV/AIDS patients attending antiretroviral therapy in public health institutions, West Shewa Zone, Ethiopia, 2017.

Clinical characteristics	Frequency	Percent (%)
WHO clinical Stage		
Stage I	49	9.7
Stage II	87	17.2
Stage III	241	47.7
Stage IV	128	25.4
Recent CD4 count		
<350 cells/*μ*l	388	76.8
≥350 cells/*μ*l	117	23.2
Duration on ART		
<44 months	261	51.7
≥44 months	244	48.3
Tuberculosis		
Yes	63	12.5
No	442	87.5
Anemia status		
Normal	401	79.4
Anemic	104	20.6
Household food security		
Secure	327	64.8
Insecure	178	35.2

**Table 3:** Nutritional status of HIV/AIDS patients attending antiretroviral therapy in public health facilities, West Shewa Zone, Ethiopia, 2017.

Variables	Undernourished		*χ* ^2^-value	*P* value
	No	Yes		
	Number (%)	Number (%)		
Age				
18–24	31 (81.6)	7 (18.4)	0.6	0.968
25–34	134 (76.6)	42 (23.4)		
35–44	146 (75.7)	47 (24.3)		
45–54	62 (75.6)	20 (24.4)		
>54	13 (76.5)	4 (23.5)		
Sex				
Male	196 (81.3)	45 (18.7)	6.8	0.009
Female	190 (72.0)	74 (28.0)		
Residence				
Rural	263 (75.1)	87 (24.9)	1.1	0.299
Urban	123 (79.3)	32 (20.7)		
Marital status				
Single	67 (72.8)	25 (27.2)	8.8	0.032
Married	263 (78.1)	74 (21.9)		
Widowed	34 (66.7)	17 (33.3)		
Divorced/separated	21 (88.0)	3 (12.0)		
Ethnicity				
Oromo	316 (75.8)	101 (24.2)	2.5	0.293
Amhara	55 (77.5)	16 (22.5)		
Others	15 (88.2)	2 (11.8)		
Religion				
Protestant	139 (78.5)	38 (21.5)	1.3	0.72
Orthodox	223 (74.6)	76 (25.4)		
Muslim	20 (83.3)	4 (16.7)		
Others	4 (80.0)	1 (20.0)		
Education				
Illiterate	126 (69.6)	55 (30.4)	7.1	0.004
Literate	260 (80.2)	64 (19.8)		
Occupation				
Employed	337 (79.2)	86 (20.8)	28.9	0.0001
Unemployed	49 (59.8)	33 (40.2)		
WHO clinical Stage				
Stage I	45 (91.8)	4 (8.2)	30.8	0.0001
Stage II	78 (89.7)	9 (10.3)		
Stage III	183 (75.9)	58 (24.1)		
Stage IV	80 (62.5)	48 (37.5)		
CD4 count				
<350 cells/*μ*l	281 (72.7)	107 (27.6)	8.7	0.003
≥350 cells/*μ*l	105 (89.7)	12 (10.3)		
Tuberculosis				
Yes	38 (60.3)	25 (39.7)	10.1	0.002
No	348 (78.7)	94 (21.3)		
ART duration				
<44 months	183 (70.1)	78 (29.9)	8.3	0.004
≥44 months	203 (73.2)	41 (16.8)		
Household food security				
Secure	295 (90.2)	32 (9.8)	118.5	0.0001
insecure	91 (51.1)	87 (48.9)		
Total	386 (76.4)	119 (23.6)		

**Table 4:** Factors associated with nutritional status of HIV/AIDS patients attending ART in public health facilities, West Shewa Zone, Ethiopia, 2017.

Variable	Undernourished		COR (95% CI)	AOR (95% CI)
	No	Yes		
	Number (%)	Number (%)		
Sex				
Male	196 (81.3)	45 (18.7)	1	1
Female	190 (72.0)	74 (28.0)	1.7 (1.1–2.6)^*∗*^	1.4 (0.8–2.3)
Marital status				
Single	67 (72.8)	25 (27.2)	1.4 (0.8–2.3)	1.3 (0.4–4.3)
Married	263 (78.1)	74 (21.9)	1	1
Widowed	34 (66.7)	17 (33.3)	1.5 (1.1–2.9)^*∗*^	1.7 (0.4–5.7)
Divorced/separated	21 (88.0)	3 (12.0)	0.7 (0.2–2.1)	0.5 (0.1–4.8)
Education				
Illiterate	126 (69.6)	55 (30.4)	1.6 (1.1–2.4)^*∗*^	1.2 (0.7–2.3)
Literate	260 (80.2)	64 (19.8)	1	1
Occupation				
Employed	337 (79.2)	86 (20.8)	1	1
Unemployed	49 (59.8)	33 (40.2)	4.1 (2.4–7.0)^*∗∗*^	3.4 (1.8–5.3)^*∗∗*^
WHO clinical stage				
Stages I/II	123 (90.4)	13 (9.6)	1	1
Stages III/IV	263 (70.5)	106 (28.8)	3.4 (1.9–6.0)^*∗∗*^	3.3 (1.8–6.5)^*∗∗*^
CD4 count				
<350 cells/*μ*l	281 (72.7)	107 (27.6)	2.6 (1.4–4.9)^*∗∗*^	2.0 (1.8–4.2)^*∗*^
≥350 cells/*μ*l	105 (89.7)	12 (10.3)	1	1
Tuberculosis				
Yes	38 (60.3)	25 (39.7)	2.5 (1.4–4.3)^*∗∗*^	2.3 (1.3–4.9)^*∗*^
No	348 (78.7)	94 (21.3)	1	1
ART duration				
<44 months	183 (70.1)	78 (29.9)	2.1 (1.4–3.2)^*∗*^	1.8 (1.2–2.9)^*∗*^
≥44 months	203 (73.2)	41 (16.8)	1	1
Food security				
Secure	297 (90.8)	30 (9.2)	1	1
Insecure	84 (47.2)	94 (52.8)	8.1 (5.9–12.8)^*∗∗*^	5.3 (2.5–8.3)^*∗∗*^

*Note.* Each adjusted odds ratio is adjusted for the remaining variables shown in the table. ART = antiretroviraltherapy; AOR = adjusted odds ratio; COR = crude odds ratio. ^*∗*^*P* value < 0.05; ^*∗∗*^*P* value < 0.01.

## Abbreviations

AIDS: Acquired Immune Deficiency Syndrome
AOR: Adjusted odds ratio
ART: Antiretroviral therapy
CI: Confidence interval
IQR: Interquartile range
PLWHA: People living with HIV/AIDS
OIs: Opportunistic infections
TB: Tuberculosis
WHO: World Health Organization.
